# Motor point heatmap of the calf

**DOI:** 10.1186/s12984-023-01152-5

**Published:** 2023-03-01

**Authors:** Elias Schriwer, Robin Juthberg, Johanna Flodin, Paul W. Ackermann

**Affiliations:** 1 Department of Molecular Medicine and Surgery, Karolinska Institutet, Stockholm, Sweden; 2 Department of Trauma, Acute Surgery and Orthopaedics, Karolinska University Hospital, Stockholm, Sweden

**Keywords:** Electric stimulation therapy, Motor point, NMES, Gastrocnemius muscle

## Abstract

**Background:**

Contractions of muscles in the calf induced by neuromuscular electrical stimulation (NMES) may prevent venous thromboembolism, help rehabilitation and optimize strength training, among other uses. However, compliance to NMES-treatment is limited by the use of suboptimal stimulation points which may cause discomfort and less effectivity. Knowledge of where one is most likely to find muscle motor points (MP) could improve NMES comfort and compliance.

**Aims:**

To anatomically map the MPs of the calf as well as to calculate the probability of finding a MP in different areas of the calf.

**Material and methods:**

On 30 healthy participants (mean age 37 years) anatomical landmarks on the lower limbs were defined. The location of the four most responsive MPs on respectively the medial and lateral head of gastrocnemius were determined in relation to these anatomical landmarks using a MP search pen and a pre-set MP search program with 3 Hz continuous stimulation (Search range:4.0–17.5 mA). The anatomy of the calves was normalized and subdivided into a matrix of 48 (6 × 8) smaller areas (3 × 3 cm), from upper medial to lower lateral, in order to calculate the probability of finding a MP in one of these areas. The probability of finding a MP was then calculated for each area and presented with a 95% confidence interval.

**Results:**

The MP heatmap displayed a higher concentration of MPs proximally and centrally on the calf. However, there were wide inter-individual differences in the location of the MPs. The highest probability of finding a MP was in area 4, located centrally and medially, and in area 29, located centrolaterally and around the maximum circumference, both with 50% probability (95% CI: 0.31–0.69). The second highest probability of finding MPs was in areas 9, 10, 16, proximally and medially, all with 47% probability (95% CI: 0.28–0.66). These areas 4, 9, 10, 16 and 29 exhibited significantly higher probability of finding motor points than all areas with a mean probability of 27% and lower (p < 0.05) The lateral and distal outskirts exhibited almost zero probability of finding MPs.

**Conclusions:**

This MP heatmap of the calf could be used to expedite electrode placement and to improve compliance in order to receive consistent and enhanced results of NMES treatments.

## Introduction

Neuromuscular electrical stimulation (NMES) is today a technology available for both clinicians and patients [1, 2]. Clinical indications include prevention of muscle atrophy during immobilization, and muscle strengthening after atrophy has occurred [1–6]. Recently calf-NMES was also demonstrated to improve venous return [7] in order to prevent venous thrombosis [8]. The efficacy and compliance to NMES treatment is, however, dependent of optimal positioning of electrodes in order to minimize discomfort [9–11].

Optimal placement of electrodes on the skin on so called motor points (MPs) has been demonstrated to substantially reduce discomfort during NMES [10–12]. The anatomical definition of a MP has for more than a hundred years been the point on the skin overlaying the location where the motor nerve enters the muscle [13–16]. The new electrophysiological definition, however, identifies a MP on the skin related to a muscle, when in combination with a reference point on the skin is exposed to electrical stimulation, and this MP-reference point combination require the least amount of electrical stimulation to induce a contraction of that specific muscle [17–20]. The location of a MP may however differ by using the electrophysiological- as compared to the anatomical definition of a MP [17].

At present, a “MP search pen” is used to localize MPs [21]. This search procedure is however time-consuming and therefore often not used in clinical practice, which leads to suboptimal electrode placements, poor patient compliance and inferior treatment effect [1].

An alternative approach to speed up the MP search or to pragmatically apply electrodes would be to use an anatomical chart of MPs, which could simplify the use of NMES in daily clinical practice. Previous studies have tried to map out MPs on different parts of the body, including the calf, by using NMES [18–20]. The prior studies and charts of MP have, however, been unspecific in anatomical details and have not been able to show the probability of finding a MP in different anatomical areas of the calf.

Therefore, we hypothesized that MPs of the calf could be mapped in a more refined manner including calculations of the probability of finding MPs in different areas. The purpose of this study was therefore to identify and describe the MPs distribution of the calf and to compute the probabilities of finding a MP on an anatomical heatmap of a calf matrix consisting of 48 (6 × 8) areas sized 3 × 3 cm.

## Materials and methods

### Study design

A total of 30 healthy subjects (19 men and 11 women) participated in the study between the 13th of April and the 9th of August 2020 performed at the Karolinska University Hospital. Healthy subjects aged between 18 and 70 years were eligible for inclusion. Healthy was defined as currently not being admitted to a hospital. All subjects signed an informed consent, completed a questionnaire on the subject characteristics (age, sex, height, weight, smoking, physical activity level (PAS) [22], Table 1) and that they did not meet any of the exclusion criteria before the study. The exclusion criteria were pregnancy, skin ulcer, pacemaker, intracardiac defibrillator, advanced heart disease, kidney failure and neuromuscular or metabolic disease. The study included MP scanning according to a predefined protocol on both the medial and lateral side of the calf.

**Table 1:** Subject characteristics

Variable	(n = 30)
Age (years) M (SD)	37.2 (13.5)
Sex (male) n (%)	19 (63)
Height (cm) M (SD)	178.3 (10.2)
Weight (kg) M (SD)	71.6 (12.2)
BMI (kg/m^2^) M (SD)	22.4 (2.7)
Side tested, left n (%)	16 (47)
PAS, range 1–6 M (SD)	5.5 (0.7)

*M* mean, *SD* standard deviation. *BMI* body mass index. *PAS* physical activity scale [22]

### Procedure

The test protocol included measurements of the calf and MP scans. Either the right or left leg was randomized for each study subject. The study subjects were instructed to lay in prone position on a gurney with the foot of hanging free outside the edge of the gurney, allowing for unrestricted movement, and thus enabling the researcher to clearly monitor plantar flexion.

**Fig. 1:**
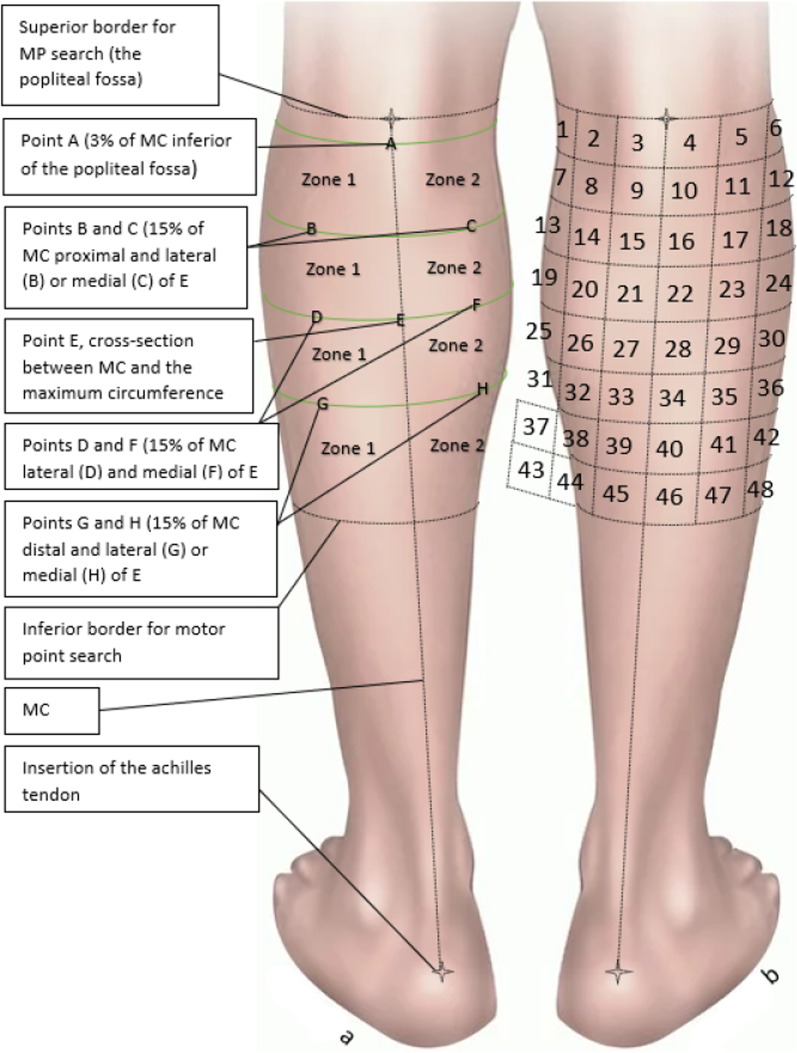
**a** Zones and reference locations of the calf. **b** Probability zones

The midline of the calf (MC) was defined as the line connecting the center of the fossa poplitea to the calcaneal insertion of the Achilles tendon (Fig. 1). Starting from the fossa poplitea, a point was marked at 3% of the distance between the two anatomical landmarks mentioned (*Reference point A)*. The choice of location for reference point A was inspired by an article by Lyons et al. [9], which used a similar reference point, located approximately 1.25 cm below the origin of the gastrocnemius muscle, with 1.25 cm roughly corresponding to 3% of the total length of the calf. A *reference point E* was marked on MC at the level of the maximum circumference of the calf.

As seen on the left calf, the midline of the Calf (MC) was defined as the distance from the midline of fossa poplitea to the calcaneal insertion of the Achilles tendon. *Reference point E* was marked on MC at the maximum circumference of the calf. Points D and F were marked out at a distance medially and laterally from E corresponding to 15% of the maximum circumference of the calf. Using that same distance from E, points B and C were marked out laterally/medially in the proximal direction and points G and H was marked out laterally/medially in the distal direction. The midline divided the calf in a medial and lateral part which were used when scanning for motor points (MP). The right calf shows the 3 × 3 cm areas later used when calculating the probability of finding a MP.

The areas in which probability was calculated. The areas only include the parts of the calf where MPs could be found. Each area (1–48) is 3 × 3 cm large, starting with nr. 1 at the most medial part of the calf, closest to fossa poplitea. and ending with nr. 48 at the lateral part of the calf.

In this study, a MP was defined as the point on the skin that resulted in a small but clearly visible contraction of a muscle in the calf at the lowest possible level of stimulation by a NMES-device, Chattanooga Theta 500 (DJO Nordic, Malmoe, Sweden). The MP scans were performed by three experienced examiners. When searching for MPs, a MP search pen (DJO Nordic, Malmoe, Sweden) was used to scan both the lateral and medial head of the gastrocnemius, one side at a time. Prior to the MP scan, subjects were familiarized with the sensation of the MP search pen stimulus. To locate the MPs, the lateral side of the calf, Zone 1 (Fig. 1), was covered with a thin layer of conductive gel. The NMES-device was pre-set to a MP scan program using 3 Hz continuous stimulation. An electrode was placed on reference point D while scanning for the lateral MPs, and reference point F for the medial MPs. The intensity (amplitude) was gradually increased on the NMES-device (0–999), which represented a non-linear scale of current ranging from 0 to 120 milliampere (mA). The MP scan was performed separately for Zone 1 (lateral side) and Zone 2 (medial side), and beginning with Zone 1, started by scanning through the entirety of the Zone being searched for a MP.If no MP was found at the the lowest NMES-level, the entirety of the same Zone would be searched with successively higher NMES-levels, until a muscle contraction could be detected, indicating the localization of a MP. The MP scan was repeated until four separate MPs had been discovered on each side (Zone 1 and 2), or until the participant choose to abort the MP scan. In this study, 4 MPs were found on each side (Zone 1 and 2) on all participants. The MPs were ranked from MP1 to MP4, where MP1 was discovered at the lowest NMES-level that could induce a muscle response, and MP4 at the 4^th^ lowest. If two MPs were discovered at the same NMES-level, the one with the most visibly clear muscle contraction was given the highest rank. MPs within a radius of 2 cm from a previously discovered MP were excluded due to difficulty in differentiating between the points. The intensities (mA) required to find MP1-4 were (mean ± SD) 6.1 ± 5.1, 7.2 ± 6.1, 8.0 ± 6.3, 8.8 ± 7.3, respectively. All MPs were found within the range, minimum–maximum, of the stimulation intensity of the search to find MP1-4 in all participants was from 4 to 17.5 mA.

### Mapping of calf anatomy and motor points

**Fig. 2:**
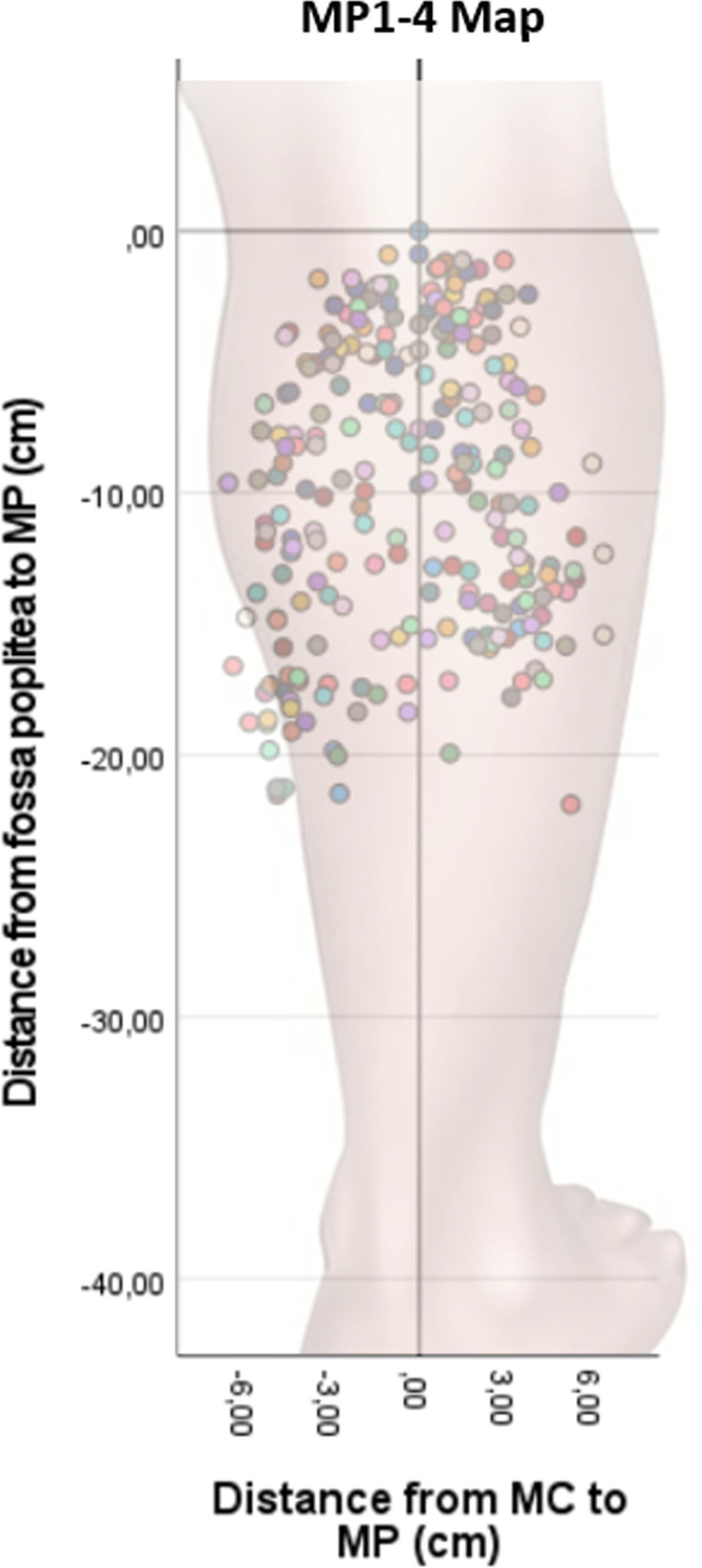
MP1-4 map. The map displays the four best MPs of 30 subjects. The colour indicates the different participants in the study. MC = midline of the calf. MP = motor point. Minus from MC = medial. Plus from MC = lateral

**Fig. 3:**
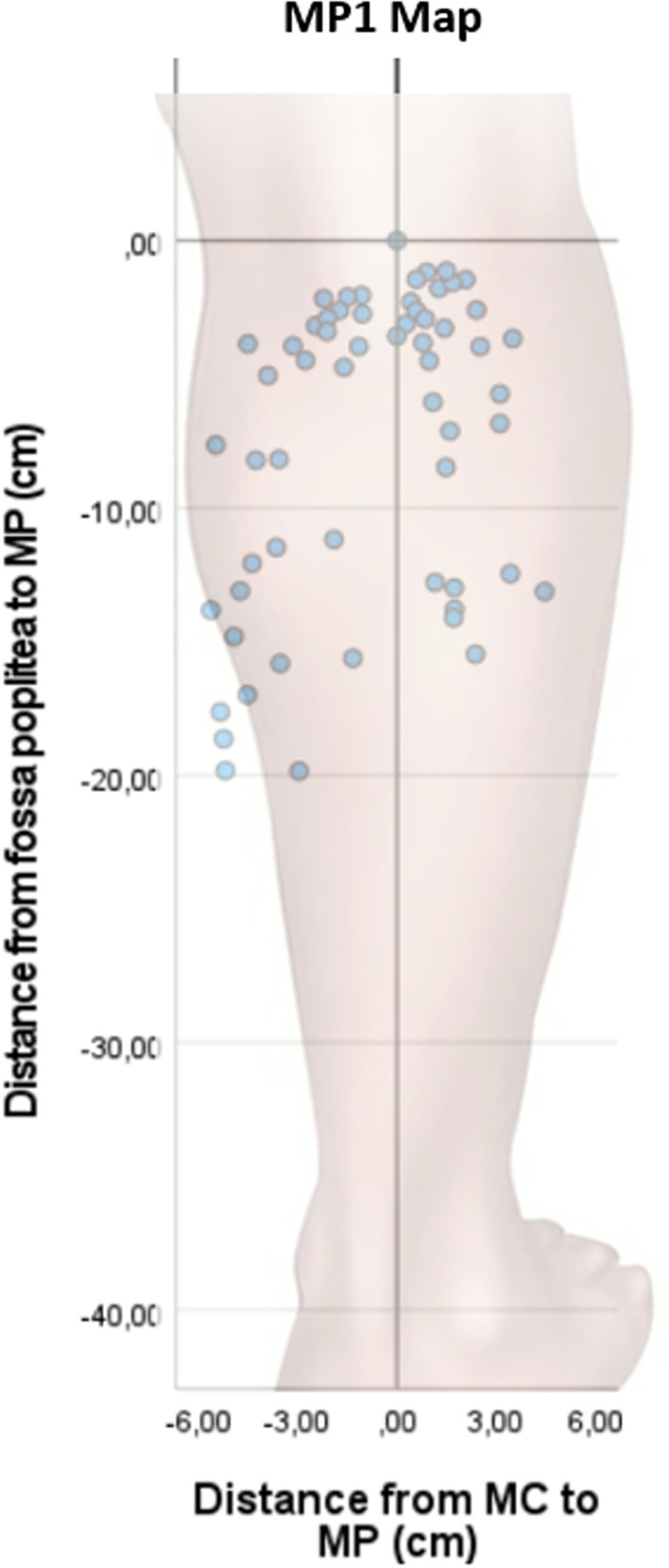
MP1 map. The map displays only the best MPs on the medial- and lateral gastrocnemius, respectively, from 30 subjects. *MC* midline of the calf. *MP* motor point. Minus from MC = medial. Plus from MC = lateral

A “normalized calf” representing the mean measurements for all 30 subjects, was created by calculating the mean distance from fossa poplitea to the insertion of the Achilles tendon, as well as the mean distance from the line of maximum calf circumference to the fossa poplitea. In order to map out the normalized locations of the MPs on the normalized calf, each subject’s actual MP location measurements relative to fossa poplitea and MC were first translated into relative percentual measurements. Subsequently these measurements were applied on the normalized calf and expressed as normalized measurements in cm (all measurements and maps mentioned hereafter are normalized). This created a map of all collected MPs regardless of rank (Fig. 2). In order to confirm our finding from the MP1-4 map we constructed a second map, which displayed only the best MPs with rank 1 (Fig. 3) on the medial- and lateral gastrocnemius head, respectively.

A third map, the MP heatmap, was created by dividing the calf into 48 (6 × 8) smaller areas (3 × 3 cm) to calculate the probability of finding a MP in each of these areas. The 48 areas, started with area 1 at the superior, medial part of the calf, and continued to area 48 at the inferior lateral part (Fig. 1b). Areas of 3 × 3 cm were chosen since pilot-tests indicated that 3 × 3 cm electrode size provides optimal balance between comfort and low NMES-level required to induce muscle contraction. This would in theory be the electrode size that would give the best compromise between comfort and battery life in a situation where long term NMES-treatment would be desirable, for example for the purpose of blood clot prevention.

### Statistics

All descriptive statistics as well as the analyzes were conducted in SPSS (Statistical Package for Social Science, IBM, version 28.0. Armonk, NY, USA). The level of significance was set p < 0.05 for all analyses. The sample size was determined prior to the start of the experiment using a power calculation based on a pilot study with the significance level set at p < 0.05 and power at 80% regarding the primary outcome, a higher probability of finding a MP in one area as compared to another area. Based on an estimated probability of 50% of finding a MP in one area and a probability of 20% of finding a MP in an adjacent area, 22 subjects were needed to obtain a significant difference. We set the final sample size to 30 subjects.

The variables were checked for severe skewed distributions using Shapiro Wilks and since no such skewness was found the descriptive data were reported as mean, standard deviation and frequency. For each test subject, each of the 48 areas described above was designated either the value 1 if it contained a MP or a 0 if it contained no MP. For each subject, each area could only be assigned the value 0 or 1, even in those few cases where one area in a subject contained more than one MP. Using SPSS, the percentual number of subjects that had received the value 1 in any given area (1–48) was calculated and presented with a 95% confidence interval, using a Clopper-Pearson test. This percentual number represented the overall probability of any given area containing one or more MPs among the subjects in our study sample.

## Results

### MP1-4 map and MP1 map

A great interindividual variation in the localization of calf muscle MPs was found on the MP1-4 map among the 30 subjects (Fig. 2). The most medial- and lateral MPs were found 6.6 cm and 6.4 cm (18% of the maximum circumference), respectively from MC. The most distal MP was found at 21.9 cm (51%) distal from fossa poplitea on the MC measuring 43.0 cm in length. The anatomical localization with the highest concentration of MPs was observed proximally close to fossa poplitea. The area with the second highest concentration of MPs was found in the vicinity of the transverse plane at the maximum circumference of the calf (Fig. 2).

Both the MP1-4 map and the MP1 map demonstrated that the two areas with the highest concentrations of MPs were discovered on a transverse line around 3 cm (7%) distal to fossa poplitea and in the vicinity of the transverse plane, around 14 cm (33%) distal to fossa poplitea, at the level of the maximum circumference of the calf (Fig. 3).

### Motor point heatmap

**Fig. 4:**
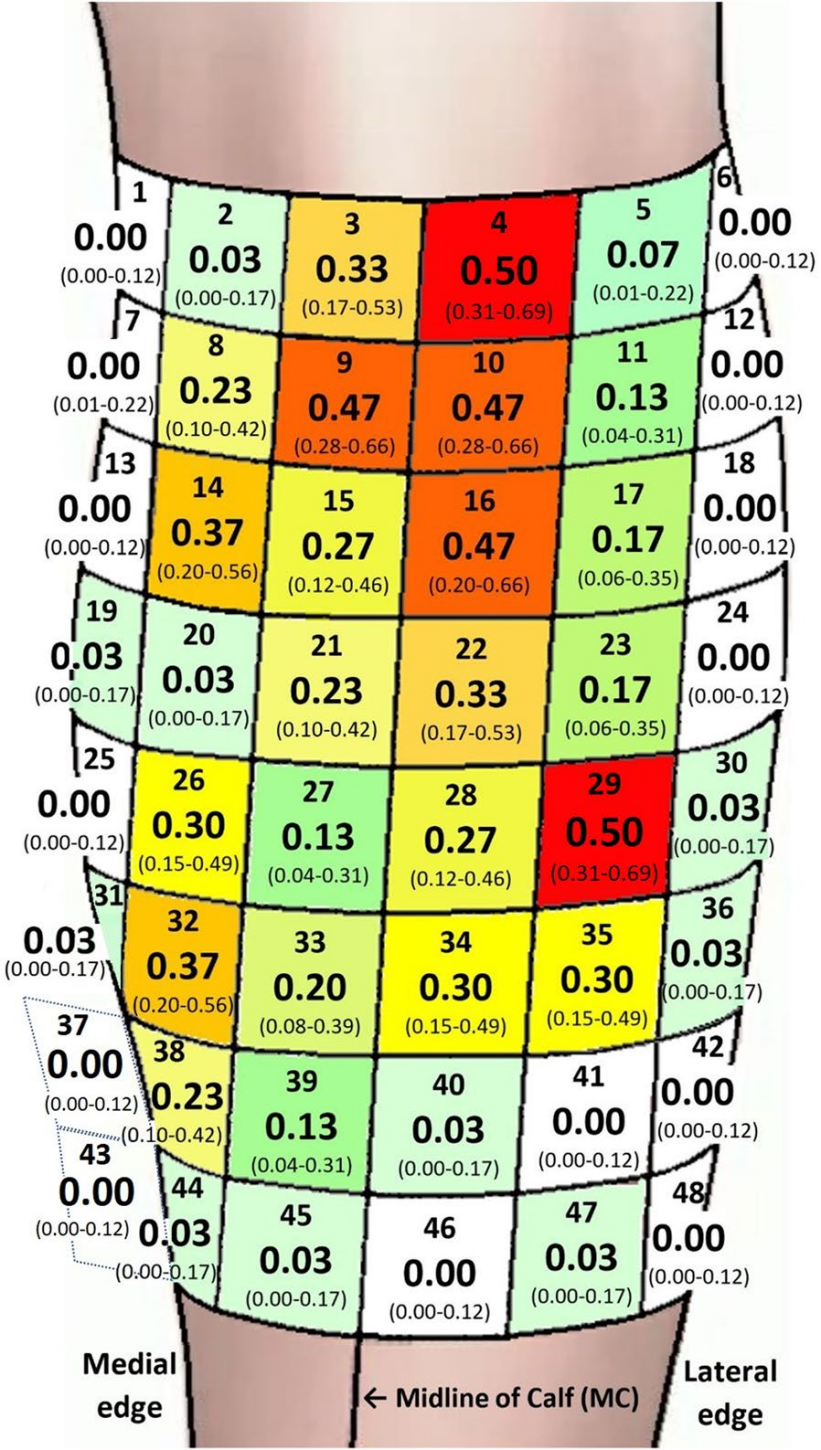
The probability of finding MPs plotted in the areas (1–48), presented with a 95% CI. The dark red areas (4,9,10,16,29) display a statistically significant higher probability of finding MPs compared to the shades of yellow, green and gray/white areas. The orange areas (14,32) display a statistically significant difference compared to the green and grey/white areas. The grey/white areas did not display a MP. *MP* Motor Point, *CI* Confidence Interval. Area 1 = medial, proximal. Area 48 = distal, lateral

In order to inform the clinician about the likelihood of finding MPs at different anatomical localizations we constructed an anatomical map, consisting of 48 (6 × 8) areas sized 3 × 3 cm, and calculated the probabilities of finding MPs in each area (MP heatmap). Overall, the areas with the lowest probabilities of finding MPs were the most medial, lateral and distal areas of the calf (Fig. 4).

The five areas (3 × 3 cm) with the highest probabilities of finding a MP resulted in around 50% chance of finding a MP (Fig. 4, shades of red). The specific localizations of the 5 best areas were found in areas 4 and 29 both with (0.50, 95% CI 0.31–0.69), and areas 9, 10 and 16 with (0.47 95% CI 0.28–0.66). These areas 4, 9, 10, 16 and 29 exhibited significantly higher probability of finding motor points than all areas with a mean probability of 0.27 and lower (p < 0.05)(shades yellow, green and gray/white).

The two areas 14 and 32 both (0.37, 95% CI 0.20–0.56), with the second highest probabilities of finding a MP resulted in 37% chance of finding a MP (Fig. 4, shades of orange). The areas 14 and 32 exhibited significantly higher probability of finding motor points than all areas with a mean probability of 0.17 and lower (p < 0.05).

The five areas with the third highest probabilities of finding a MP were observed in areas 3 and 22 (0.33 95% CI 0.17–0.53), and areas 26, 34 and 35 (0.30, 95% CI 0.15–0.49). These areas 3, 22, 26, 34 and 35 exhibited significantly higher probability of finding motor points than all areas with a mean probability of 0.13 and lower (p < 0.05). Neither of the areas in grey shades; 1, 6, 7, 12, 13, 18, 24, 25, 37, 41, 42, 43, 46 or 48, contained any MPs.

## Discussion

This study demonstrated a pronounced inter-individual variation in the localization of calf muscle MPs. However, the two areas with the highest concentrations of MPs were localized in the midline close to fossa poplitea and in the proximity of the transverse plane at the maximum circumference of the calf. A computed MP heatmap displayed the probabilities of finding MPs on an anatomical chart, which may guide and help the clinician in daily NMES application.

The prominent inter-individual variability in the anatomical localization of MPs on the calf muscle was the main finding of this study, which suggests that each patient, when possible, should undergo a MP scan before NMES electrodes are applied. The MP heatmap will give suggestions on where to start a MP scan in order to reduce the time of scanning. If a MP scanning device is not available, the heatmap suggests areas to place the electrodes. The observation of a high inter-individual variability in MP localization is corroborated by earlier research mainly of cadaveric studies on M. triceps surae, but also of one study on MP locations of the gastrocnemius muscle [14, 15, 19]. To the best of our knowledge, however, this is the first study, which in a detailed and focused manner has successively searched for and identified the four best MPs in each subject examined on respectively the lateral and medial head of gastrocnemius.

The observation of the most distal MP being localized at around 50% length of the calf suggests that clinicians do not have to scan for MPs below the mid of the calf when applying calf NMES. This finding is strengthened by an earlier cadaveric study by Kim et al. from 2005, who found that the most distal MP was identified at around 40% of the calf length distal from fossa poplitea [15]. Interestingly, however, NMES treatment may induce multiple molecular adaptations including axonal sprouting and newly formed neuromuscular junctions, suggesting that MP locations may change which warrants further studies [23].

Our identification of the most medial- and lateral MPs both being localized at around 6 cm (18%) from the MC implies a further restriction of the search area for the clinician performing the MP scan. The observation is supported by another cadaveric study by Sook Kim et al. who found the main MPs on the medial and lateral gastrocnemius at around one third and two third of the individual calf width divided with 2, respectively, from the central aspect of the calf [14].

The second main finding of this study was the localization of the two areas on the calf that exhibited the highest concentrations of MPs. The observation of one area of concentration of MPs close to the midline and some centimetres distal to fossa poplitea, would seem to be substantiated by earlier studies [19]. The earlier studies did however not clearly present their suggested MP-locations in relation to a system of areas with predefined size, thus making their results harder to apply in clinical practice. In contrast, our presentation is based on specific anatomical landmarks and provides the clinician with an easy-to-use chart display, based on probabilities of a statistical significance, on where to start looking for the MPs.

The identified popliteal concentration of MPs, directly distal of fossa poplitea, may reflect the branching of the tibial motor nerve, which supplies the lateral and medial gastrocnemius. The other high concentration of MPs found at the maximum circumference of the calf in the proximity of the transverse plane has not been identified in earlier cadaveric studies [14, 15]. Botter et al. who also used a MP scan, however identified MPs close to the maximum circumference of the calf, which would seem to support our findings [19]. The higher concentration of MPs at the maximum circumference of the calf both on the medial and lateral gastrocnemius head is therefore new knowledge. The difference in MP-location between the previous cadaveric studies and MP scan studies are likely an effect of the differing methods used to define the MPs, as has been suggest earlier [17]. Whereas the cadaveric studies defined the MPs as the location where the nerve entered the muscle, modern MP scan studies defined the MP-locations as the points on the skin that most readily induced a muscle response with NMES.

Based on our main findings we established a novel MP heatmap displaying the probabilities of finding MPs in 48 predefined, 3 × 3 cm areas, of the calf. The heatmap displayed that central and proximal areas of the calf showed a much greater probability of containing a MP than the most medial, lateral, and distal areas, which confirmed the findings of the MP1-4 map. The graphical display of the heatmap moreover suggests an anatomical chart, which can be used to search for MPs in a subsequent, logical order [1].

The observation of two areas on the calf, number 4 and 29, demonstrating the highest probability of containing a MP at 50%, suggests that these areas may be good starting points for the clinician to locate MPs. Area 4 located 0–3 cm below fossa poplitea and just lateral of the midline, and area 29, located 12–15 cm below fossa poplitea at the largest circumference of the calf and 3–6 cm lateral of the midline, may be easily identified by the clinician. The finding that areas 4 and 29 exhibited significantly higher probability of identifying a MP than in 39 of the 48 areas examined on the calf strengthen the observation that these areas are good starting points to look for MPs.

The discovery that the three areas, 9, 10 and 16, which demonstrated the second highest probability of finding a MP, 47%, shared border with each other and with area 4, implies how a manual search with MP-pens on the calf could be performed. After the search in area 4 we suggest that the search of MPs should continue in a distal and medial direction to are 9, followed by searching laterally towards area 10, subsequently in a distal direction to area 16. Then continuing the search distally towards area 29, passing areas 22 and 28 on the way.

One disadvantage with the search algorithm described so far is that only area 9 represents the medial gastrocnemius head. This is however in line with earlier research that has also experienced problems in finding MPs on the medial gastrocnemius head [19]. If area 9 did not result in finding a MP on medial gastrocnemius we recommend proceeding with the two areas with the third highest probability of finding a MP, 37%, areas 14 and 32. After examining area 9 the clinician should continue medially and distally to area 14, and subsequently 6 cm distal to reach area 32. The search algorithm described so far is of course not unique, but is rather a suggestion on how to best use the MP heatmap in order to locate the MPs of the calf in the most time efficient and optimized way.

A potential weakness of the study is that we cannot with any statistical significance say that there is a difference in probability of finding a MP between the areas that displayed higher probability than 33%. This finding, however, suggests that the two best areas have significantly higher likelihood of containing a MP than all areas with 30% probability, which is a strength of the study. Similarly, the areas with the second highest probabilities of 47%, exhibit significantly better chances of containing a MP compared to the areas with 27% probability and lower, which confirms the benefit of using the heatmap. Another limitation is that some MPs were discarded when calculating the heatmap, since they were situated in the same area as another MP. However, the discarded MPs were very few, and did not affect the result in any major way.

## Conclusions

This study demonstrated a distinct inter-individual variation in the localization of MPs on the calf muscle. However, certain anatomical patterns of likelihood of where to find MPs seem to repeat among subjects. A heatmap revealed the probabilities of finding MPs in different anatomical areas, suggesting a successive algorithm, which may help clinicians when searching for MPs in daily practice.

## Data Availability

The datasets used and/or analyzed during the current study are available from the corresponding author on reasonable request.

## Abbreviations

DVT: Deep vein thrombosis
IPC: Intermittent pneumatic compression
MP: Motor point
NMES: Neuromuscular electrical stimulation
PE: Pulmonary embolism
MC: Midline of the calf
